# Supplementary material for: Modeling glioblastoma heterogeneity as a dynamic network of cell states
Source: Mol Syst Biol. 2021 Sep 16;17(9):e10105. doi: 10.15252/msb.202010105 (PMC8444284; doi:10.15252/msb.202010105)
Supplement: Supplementary file 6 — Source Data for Figure 5 [file MSB-17-e10105-s004.zip › Figure5A_sourcedata/GSEA_3071/nosigdb_stateIV.GseaPreranked.1622795164626/RICHARDS_VERHAAK_CANCERCELL_2010_MESENCHYMAL.html]

Details for gene set RICHARDS\_VERHAAK\_CANCERCELL\_2010\_MESENCHYMAL[GSEA]

|  || Dataset | state53071 |
| Phenotype | NoPhenotypeAvailable |
| Upregulated in class | na\_neg |
| GeneSet | RICHARDS\_VERHAAK\_CANCERCELL\_2010\_MESENCHYMAL |
| Enrichment Score (ES) | -0.54676825 |
| Normalized Enrichment Score (NES) | -2.925724 |
| Nominal p-value | 0.0 |
| FDR q-value | 0.0 |
| FWER p-Value | 0.0 |
Table: GSEA Results Summary

  

Fig 1: Enrichment plot: RICHARDS\_VERHAAK\_CANCERCELL\_2010\_MESENCHYMAL      
 Profile of the Running ES Score & Positions of GeneSet Members on the Rank Ordered List

  

| PROBE | GENE SYMBOL | GENE\_TITLE | RANK IN GENE LIST | RANK METRIC SCORE | RUNNING ES | CORE ENRICHMENT || 1 | PDPN |  |  | 76 | 1.689 | 0.0038 | No |
| 2 | COL1A2 |  |  | 137 | 1.425 | 0.0078 | No |
| 3 | WWTR1 |  |  | 277 | 1.081 | -0.0072 | No |
| 4 | HEXA |  |  | 315 | 1.018 | -0.0032 | No |
| 5 | FNDC3B |  |  | 661 | 0.681 | -0.0623 | No |
| 6 | SAT1 |  |  | 859 | 0.575 | -0.0940 | No |
| 7 | MRC2 |  |  | 969 | 0.535 | -0.1092 | No |
| 8 | MAFB |  |  | 1248 | 0.452 | -0.1579 | No |
| 9 | EFEMP2 |  |  | 1311 | 0.438 | -0.1651 | No |
| 10 | ITGA4 |  |  | 1355 | 0.427 | -0.1687 | No |
| 11 | SYNGR2 |  |  | 1577 | 0.371 | -0.2073 | No |
| 12 | PTGER4 |  |  | 1797 | 0.331 | -0.2459 | No |
| 13 | FCGR2A |  |  | 2054 | 0.289 | -0.2922 | No |
| 14 | TNFRSF1A |  |  | 2234 | 0.264 | -0.3238 | No |
| 15 | TLR4 |  |  | 2287 | 0.258 | -0.3310 | No |
| 16 | IL15RA |  |  | 2354 | -0.253 | -0.3410 | No |
| 17 | CLCF1 |  |  | 2356 | -0.254 | -0.3384 | No |
| 18 | SLC10A3 |  |  | 2429 | -0.261 | -0.3495 | No |
| 19 | MGAT1 |  |  | 2460 | -0.264 | -0.3524 | No |
| 20 | RBKS |  |  | 2575 | -0.279 | -0.3713 | No |
| 21 | SWAP70 |  |  | 2578 | -0.279 | -0.3686 | No |
| 22 | UCP2 |  |  | 2602 | -0.283 | -0.3700 | No |
| 23 | POLD4 |  |  | 2676 | -0.292 | -0.3809 | No |
| 24 | RELB |  |  | 2850 | -0.313 | -0.4108 | No |
| 25 | SH2B3 |  |  | 2880 | -0.317 | -0.4130 | No |
| 26 | LY96 |  |  | 2935 | -0.323 | -0.4198 | No |
| 27 | FURIN |  |  | 2938 | -0.324 | -0.4167 | No |
| 28 | LAMB1 |  |  | 3029 | -0.336 | -0.4304 | No |
| 29 | ELF4 |  |  | 3036 | -0.337 | -0.4279 | No |
| 30 | CYBRD1 |  |  | 3069 | -0.341 | -0.4303 | No |
| 31 | LCP1 |  |  | 3076 | -0.342 | -0.4277 | No |
| 32 | CASP1 |  |  | 3204 | -0.360 | -0.4483 | No |
| 33 | S100A13 |  |  | 3219 | -0.363 | -0.4470 | No |
| 34 | PHF11 |  |  | 3224 | -0.364 | -0.4438 | No |
| 35 | TNFAIP3 |  |  | 3248 | -0.368 | -0.4442 | No |
| 36 | HEXB |  |  | 3270 | -0.370 | -0.4442 | No |
| 37 | TMBIM1 |  |  | 3366 | -0.385 | -0.4584 | No |
| 38 | FHOD1 |  |  | 3405 | -0.392 | -0.4614 | No |
| 39 | PIGP |  |  | 3508 | -0.411 | -0.4766 | No |
| 40 | VDR |  |  | 3524 | -0.414 | -0.4750 | No |
| 41 | TRIM22 |  |  | 3563 | -0.421 | -0.4777 | No |
| 42 | COL8A2 |  |  | 3596 | -0.428 | -0.4792 | No |
| 43 | ITGB2 |  |  | 3649 | -0.435 | -0.4845 | No |
| 44 | LGALS1 |  |  | 3692 | -0.443 | -0.4877 | No |
| 45 | RAB27A |  |  | 3744 | -0.454 | -0.4926 | No |
| 46 | RHOG |  |  | 3811 | -0.469 | -0.5002 | No |
| 47 | ITGA5 |  |  | 3864 | -0.480 | -0.5050 | No |
| 48 | SCPEP1 |  |  | 3903 | -0.487 | -0.5070 | No |
| 49 | IQGAP1 |  |  | 3939 | -0.495 | -0.5083 | No |
| 50 | DCBLD2 |  |  | 4038 | -0.521 | -0.5216 | No |
| 51 | RUNX2 |  |  | 4120 | -0.545 | -0.5312 | No |
| 52 | TRIM38 |  |  | 4143 | -0.553 | -0.5294 | No |
| 53 | ICAM3 |  |  | 4157 | -0.556 | -0.5259 | No |
| 54 | PLBD1 |  |  | 4237 | -0.576 | -0.5348 | No |
| 55 | CASP4 |  |  | 4253 | -0.582 | -0.5313 | No |
| 56 | NPC2 |  |  | 4334 | -0.608 | -0.5401 | Yes |
| 57 | PYGL |  |  | 4349 | -0.614 | -0.5361 | Yes |
| 58 | RAB32 |  |  | 4398 | -0.634 | -0.5384 | Yes |
| 59 | LGALS3 |  |  | 4418 | -0.641 | -0.5351 | Yes |
| 60 | BNC2 |  |  | 4450 | -0.654 | -0.5339 | Yes |
| 61 | RAB11FIP1 |  |  | 4508 | -0.684 | -0.5374 | Yes |
| 62 | ASL |  |  | 4518 | -0.688 | -0.5316 | Yes |
| 63 | BLVRB |  |  | 4533 | -0.698 | -0.5267 | Yes |
| 64 | RRAS |  |  | 4549 | -0.703 | -0.5219 | Yes |
| 65 | FHL2 |  |  | 4576 | -0.723 | -0.5190 | Yes |
| 66 | LTBP1 |  |  | 4661 | -0.769 | -0.5268 | Yes |
| 67 | IGFBP6 |  |  | 4693 | -0.790 | -0.5242 | Yes |
| 68 | MAN1A1 |  |  | 4710 | -0.801 | -0.5185 | Yes |
| 69 | SEC24D |  |  | 4730 | -0.815 | -0.5133 | Yes |
| 70 | YAP1 |  |  | 4744 | -0.824 | -0.5068 | Yes |
| 71 | ARPC1B |  |  | 4771 | -0.840 | -0.5026 | Yes |
| 72 | CNN2 |  |  | 4774 | -0.842 | -0.4938 | Yes |
| 73 | DSE |  |  | 4778 | -0.843 | -0.4851 | Yes |
| 74 | ANXA4 |  |  | 4817 | -0.882 | -0.4828 | Yes |
| 75 | CEBPB |  |  | 4835 | -0.902 | -0.4762 | Yes |
| 76 | SHC1 |  |  | 4885 | -0.948 | -0.4753 | Yes |
| 77 | SLC16A3 |  |  | 4918 | -0.983 | -0.4708 | Yes |
| 78 | CTSB |  |  | 4948 | -1.012 | -0.4653 | Yes |
| 79 | EMP3 |  |  | 4960 | -1.029 | -0.4562 | Yes |
| 80 | CLIC1 |  |  | 4964 | -1.035 | -0.4454 | Yes |
| 81 | LRRFIP1 |  |  | 4985 | -1.069 | -0.4376 | Yes |
| 82 | ANXA2 |  |  | 4997 | -1.087 | -0.4278 | Yes |
| 83 | COPZ2 |  |  | 5071 | -1.227 | -0.4285 | Yes |
| 84 | PROCR |  |  | 5072 | -1.228 | -0.4151 | Yes |
| 85 | UAP1 |  |  | 5073 | -1.228 | -0.4017 | Yes |
| 86 | SP100 |  |  | 5084 | -1.252 | -0.3899 | Yes |
| 87 | SRPX2 |  |  | 5085 | -1.252 | -0.3762 | Yes |
| 88 | TIMP1 |  |  | 5086 | -1.257 | -0.3625 | Yes |
| 89 | RBMS1 |  |  | 5089 | -1.263 | -0.3490 | Yes |
| 90 | CAST |  |  | 5093 | -1.274 | -0.3357 | Yes |
| 91 | S100A11 |  |  | 5100 | -1.292 | -0.3227 | Yes |
| 92 | TGFBI |  |  | 5104 | -1.305 | -0.3090 | Yes |
| 93 | P4HA2 |  |  | 5127 | -1.346 | -0.2986 | Yes |
| 94 | TNFAIP8 |  |  | 5130 | -1.363 | -0.2841 | Yes |
| 95 | MYH9 |  |  | 5132 | -1.366 | -0.2693 | Yes |
| 96 | LOX |  |  | 5149 | -1.418 | -0.2569 | Yes |
| 97 | SERPINE1 |  |  | 5162 | -1.501 | -0.2428 | Yes |
| 98 | NRP1 |  |  | 5177 | -1.553 | -0.2285 | Yes |
| 99 | FXYD5 |  |  | 5185 | -1.597 | -0.2124 | Yes |
| 100 | S100A4 |  |  | 5189 | -1.605 | -0.1955 | Yes |
| 101 | MYOF |  |  | 5193 | -1.623 | -0.1783 | Yes |
| 102 | PLAUR |  |  | 5213 | -1.721 | -0.1631 | Yes |
| 103 | THBS1 |  |  | 5222 | -1.800 | -0.1450 | Yes |
| 104 | CLEC2B |  |  | 5248 | -2.067 | -0.1272 | Yes |
| 105 | DAB2 |  |  | 5250 | -2.114 | -0.1043 | Yes |
| 106 | COL5A1 |  |  | 5276 | -2.605 | -0.0807 | Yes |
| 107 | ARHGAP29 |  |  | 5279 | -2.693 | -0.0516 | Yes |
| 108 | COL1A1 |  |  | 5290 | -4.897 | -0.0000 | Yes |
Table: GSEA details [plain text format]

  

Fig 2: RICHARDS\_VERHAAK\_CANCERCELL\_2010\_MESENCHYMAL: Random ES distribution      
 Gene set null distribution of ES for **RICHARDS\_VERHAAK\_CANCERCELL\_2010\_MESENCHYMAL**
